# Modulation of RAB7A Protein Expression Determines Resistance to Cisplatin through Late Endocytic Pathway Impairment and Extracellular Vesicular Secretion

**DOI:** 10.3390/cancers11010052

**Published:** 2019-01-08

**Authors:** Flora Guerra, Aurora Paiano, Danilo Migoni, Giulia Girolimetti, Anna Myriam Perrone, Pierandrea De Iaco, Francesco Paolo Fanizzi, Giuseppe Gasparre, Cecilia Bucci

**Affiliations:** 1 Department of Biological and Environmental Sciences and Technologies (DiSTeBA), University of Salento, Via Provinciale Lecce-Monteroni 165, 73100 Lecce, Italy; guerraflora@gmail.com (F.G.); paianoaurora@hotmail.it (A.P.); danilo.migoni@unisalento.it (D.M.); francescopaolo.fanizzi@unisalento.it (F.P.F.); 2 Department of Medical and Surgical Sciences (DIMEC), Medical Genetics Unit, University Hospital S. Orsola-Malpighi, via Massarenti 9, 40138 Bologna, Italy; giulsgiuls85@gmail.com (G.Gi.); giuseppe.gasparre3@unibo.it (G.Ga.); 3 Unit of Oncologic Gynecology, S. Orsola-Malpighi Hospital, via Massarenti 13, 40138 Bologna, Italy; myriam.perrone@aosp.bo.it (A.M.P.); pierandrea.deiaco@aosp.bo.it (P.D.I.)

**Keywords:** cisplatin, RAB7A, chemoresistance, lysosome, endocytosis

## Abstract

Background: Cisplatin (CDDP) is widely used in treatment of cancer, yet patients often develop resistance with consequent therapeutical failure. In CDDP-resistant cells alterations of endocytosis and lysosomal functionality have been revealed, although their causes and contribution to therapy response are unclear. Methods: We investigated the role of RAB7A, a key regulator of late endocytic trafficking, in CDDP-resistance by comparing resistant and sensitive cells using western blotting, confocal microscopy and real time PCR. Modulation of RAB7A expression was performed by transfection and RNA interference, while CDDP sensitivity and intracellular accumulation were evaluated by viability assays and chemical approaches, respectively. Also extracellular vesicles were purified and analyzed. Finally, correlations between RAB7A and chemotherapy response was investigated in human patient samples. Results: We demonstrated that down-regulation of RAB7A characterizes the chemoresistant phenotype, and that RAB7A depletion increases CDDP-resistance while RAB7A overexpression decreases it. In addition, increased production of extracellular vesicles is modulated by RAB7A expression levels and correlates with reduction of CDDP intracellular accumulation. Conclusions: We demonstrated, for the first time, that RAB7A regulates CDDP resistance determining alterations in late endocytic trafficking and drug efflux through extracellular vesicles.

## 1. Introduction

Cisplatin (CDDP) is a well-known platinum coordination compound used as a drug in cancer therapy usually in combination with other drugs. The platinum ions form bonds with purine bases in DNA, affecting DNA repair mechanisms and inducing apoptosis in cancer cells [1]. CDDP is effective against different types of cancer, and it has been widely used against ovary, testicular, head and neck, bladder and lung cancer. However, side effects such as nephrotoxicity and peripheral neurotoxicity, and intrinsic or acquired resistance greatly limit its use [2]. For these reasons, numerous platinum derivatives have been developed, although their success has been restricted to few compounds [2]. The main obstacle to an effective reduction of toxicity and resistance is the complexity, and the still limited knowledge, of uptake, efflux, trafficking and mechanisms of biodistribution and action of CDDP and platinum derivatives [1,3].

CDDP enters cells mainly by two different routes: passive diffusion or facilitated and active uptake through several different membrane proteins and channels [4]. Among CDDP transporters there are Cu-transporters (CTR1/2) and solute carriers (SLC), in particular organic cation transporters (OCT) [5]. Also, Na+-dependent glucose transport and other ATP-dependent transport processes have been suggested for CDDP [6,7]. Obviously, it is important to define precisely the pathways of CDDP trafficking in order to unravel causes of toxicity and resistance. Indeed, in CDDP-resistant cells a strong reduction of intracellular platinum concentration (up to 80%) has been observed, which is probably a result of reduced uptake and/or increased efflux [1,3]. Importantly, several studies have suggested that also the endocytic pathway is important for CDDP resistance [8,9,10]. In fact, CDDP resistance is accompanied by alterations of endocytosis, autophagic flux and lysosomal functionality [11,12]. Autophagic flux, for instance, promotes CDDP resistance in human ovarian carcinoma cells [13] while reduced endocytosis and altered lysosomal function have been detected in CDDP resistant cells [11,12,14]. Furthermore, the marine alkaloid monanchocidin A, which causes lysosomal membrane permeabilization, has been shown to overcome CDDP resistance [15]. However, the molecular mechanism underlying CDDP resistance is still not well understood and how endocytosis and lysosomal impairment alter CDDP resistance warrants investigation.

Notably, altered expression of RAB proteins, small GTPases regulating vesicular trafficking [16], has been correlated with CDDP resistance [9,17]. Reduced expression of RAB5A was found in CDDP-resistant epidermoid carcinoma cells, while increased RAB8A expression was detected as an early event of CDDP resistance [9,17]. Accordingly, cells overexpressing RAB8A became twice more resistant to CDDP compared to control cells [17].

RAB7A is a ubiquitous member of the RAB GTPase family localized to late endosomes and lysosomes and characterized, as all GTPases, by a mechanism of activation and inactivation depending on GTP binding and hydrolysis [18]. To study the function of RAB7A, a dominant negative mutant (RAB7A T22N) with impaired nucleotide exchange thus locking the protein in its inactive GDP-bound form, and a constitutively active mutant (Q67L) with impaired GTP hydrolysis, have been extensively used [18]. RAB7A is a key regulator of the late endocytic pathway, being important in controlling early and late endosome maturation, lysosomal biogenesis and acidification, and also clustering and fusion of late endosomes and lysosomes in the perinuclear region [19,20,21,22,23]. The acquisition of RAB7A on late endosomes is accompanied by the loss of RAB5, a marker of early sorting endosomes [24,25]. Furthermore, GTP-bound RAB7 mediates the attachment of late endosomes and lysosomes to the dynein–dynactin complex through the recruitment of RILP (Rab Interacting Lysosomal Protein) for the movement of late endosomes and lysosomes toward the microtubule organizing center [26,27,28]. In fact, expression of the RAB7A T22N dominant negative mutant causes dispersal of late endosomes and lysosomes blocking cargo trafficking to lysosomes, while expression of the RAB7A constitutively active Q67L mutant or overexpression of the wild-type protein cause the formation of large endocytic structures densely packed in the perinuclear region [19]. Expression of the RAB7A dominant negative T22N mutant compromises also lysosomal acidification because RAB7A, through its effector RILP, interacts with the V1G1 subunit of the vacuolar ATPase, controlling assembly and function of the V-ATPase on Rab7-positive organelles [29,30]. Furthermore, it has been recently demonstrated that regulation of lysosomal pH controlled by Rab7 and RILP depends also on lysosomal positioning [31]. In addition, RAB7A has a key role in phagocytosis and autophagy controlling fusion of phagosomes and autophagosomes with lysosomes [22,32,33]. Importantly, RAB7A functions impact also on many other physiological processes, such as, for instance, apoptosis, neurotrophin trafficking and signaling, neurite outgrowth, mitophagy and lipophagy [34,35,36,37,38,39]. Thus, in view of the pivotal role of RAB7A in lysosome biogenesis and function, as well as in channel and membrane protein trafficking, we thought of it as a good candidate to act as a regulator of CDDP-resistance.

We have hence investigated the involvement of RAB7A in CDDP-resistance using CDDP-resistant and sensitive cells. We demonstrated that RAB7A is downregulated in resistant cells and that RAB7A silencing increases CDDP resistance, whereas RAB7A overexpression decreases it. Moreover, although preliminary, we observed correlations between expression levels of RAB7A and chemoresistance ex vivo. Altogether, these data indicate that CDDP-resistance can be modulated by altering RAB7A expression.

## 2. Results

### 2.1. CDDP-Resistant Cells Display Alterations of Late Endocytic Pathway

**Figure 1:**
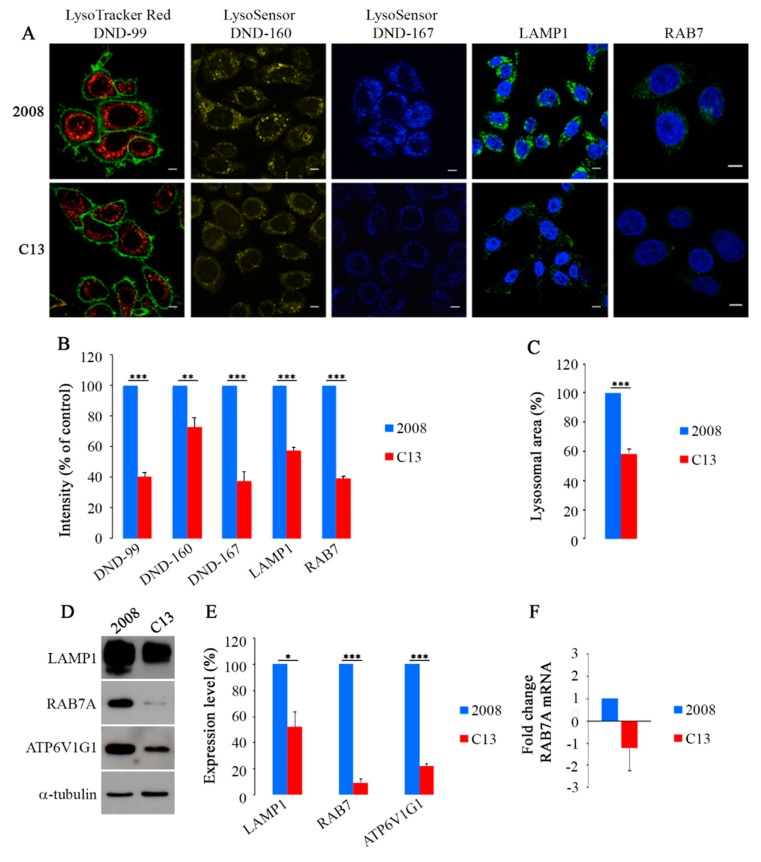
(**A**) Cells were labeled live with Lysotracker Red DND-99 and Cell Mask (green), with LysoSensor DND-160 (yellow) and with LysoSensor DND-167 (blue) or were fixed and immunostained with anti-LAMP1 and anti-RAB7A antibodies (green). Nuclei were labeled with 4’,6-diamidino-2-phenylindole (DAPI) (blue). Scale bar: 10 μm. (**B**) IF intensity and (**C**) lysosomal area were quantified by ImageJ software. Data represent the mean ± standard error (SE) (three independent experiments, ≥50 cells). (**D,E**) Relative protein abundance of LAMP1, RAB7A, ATP6V1G1 was assessed by Western Blot (WB) and quantified by densitometry normalizing against α-tubulin. (**F**) qRT-PCR was performed on 2008 and C13 cells and the amount of RAB7A mRNA was quantified relative to GAPDH. Data represent the mean ± SE of at least three independent experiments (* *p* ≤ 0.05 and ** *p* ≤ 0.01 *** *p* ≤ 0.001).

Previous data indicate that CDDP-resistant cells have altered lysosomal abundance and morphology [11,12]. To start investigating the causes of lysosomal changes induced by CDDP-resistance, we monitored distribution of lysosomes in CDDP-sensitive 2008 cells and in their CDDP-resistant counterpart C13 using vital dyes that have amine groups which are partially protonated at neutral pH and fully protonated at acidic pH and that are used to stain acidic organelles (Figure 1A) [40]. C13 cells showed a weaker staining with LysoTracker Red DND-99, compared to 2008 cells and intensity quantification demonstrated a statistically significant decrease of about 60% (Figure 1A,B). As LysoTracker fluorescence is largely pH-independent, we used also LysoSensor DND-160 and DND-167 dyes that are able to detect compartments within the pH range of 3.5–6.0 and 4.5–6.0, respectively [40]. Both staining methods confirmed that C13 cells have a significant reduction of acidic compartments compared to 2008 chemosensitive cells (Figure 1A). Indeed, intensity quantification revealed that C13 cells have about 35% and 63% reduction of LysoSensor DND-160 and DND-167 staining, respectively, compared to 2008 cells (Figure 1B). Interestingly, in chemoresistant C13 cells, we observed also reduction of about 40% in the size of labeled organelles compared to 2008 cells (Figure 1A,C).

Considering the central role of RAB7A in the endocytic pathway and in particular in lysosomal biogenesis [18,19], we decided to investigate its potential role in chemoresistance, hypothesizing that, if lysosomes of chemoresistant cells were defective, it could be due to altered RAB7A expression and function. Thus, we have performed immunofluorescence (IF) using specific antibodies against RAB7A and the lysosomal-associated membrane protein 1 (LAMP1). In C13 compared to control 2008 cells, we observed a significant reduction of both RAB7A and LAMP1 of about 60% and 43%, respectively (Figure 1A,B) and a more peripheral distribution of LAMP1. In order to confirm such decrease, we performed a western blotting (WB) analysis that revealed a strong decrease in CDDP-resistant C13 cells of about 50% and 90% in the abundance of LAMP1 and RAB7A, respectively (Figure 1D,E).

RILP, an effector of RAB7A controlling late endocytic trafficking and multivesicular bodies (MVBs) formation [26,41,42], regulates endocytic pH by controlling assembly and activity of the V-ATPase on RAB7A-positive organelles through interaction with the ATP6V1G1 subunit [29,30]. Interestingly, also ATP6V1G1 abundance was strongly decreased of about 80% in CDDP-resistant C13 cells compared to controls (Figure 1D,E). To establish whether the observed changes in the amount of RAB7A were a consequence of mRNA abundance, we used qRT-PCR to monitor mRNA and we did not find significant differences between 2008 and C13 cells, suggesting that regulation of RAB7 abundance is not transcriptional (Figure 1F). Altogether these results indicate that in chemoresistant C13 cells abundance of late endosomal and lysosomal markers, such as RAB7A, LAMP1 and ATP6V1G1 is strongly decreased, suggesting that the late endocytic pathway is altered.

In parallel, LysoTracker DND-99 and LysoSensor DND-167 staining was performed also on A431, Hela and their resistant counterparts A431 platinum (Pt) and HeLa Pt. Consistently with the results obtained with C13 and 2008 cells, a strong reduction of the staining was observed in chemoresistant A431 Pt and HeLa Pt cells compared to control A431 and HeLa cells, respectively (Figure S1A–C). Moreover, LAMP1 staining in HeLa Pt revealed a reduction of about 35% compared to control cells (Figure S1A,C). In contrast, the limited decrease of LAMP1 intensity in A431 Pt cells was not statistically significant, although intracellular distribution of LAMP1 positive organelles looked less perinuclear compared to sensitive cells similarly to C13 cells (Figure S1A,B). Also, evaluation of RAB7A abundance by IF and WB analysis showed a significant reduction of intensity both in A431 Pt and HeLa Pt cells compared to their sensitive counterparts (Figure S1B,C and S1D,E respectively). Similarly to C13 cells, qRT-PCR did not revealed significant changes in RAB7A mRNA levels (Figure S1F).

In order to understand if changes in chemoresistant cells are specific for RAB7A and the late endocytic pathway or involve also the early endocytic pathway, we decided to evaluate the expression of RAB4A and RAB5A. RAB5A controls homotypic fusion and motility of early sorting endosomes, while RAB4A regulates the exit of constitutive recycling cargo from early sorting endosomes directly back to the plasma membrane as well as to recycling compartment [16]. In contrast to RAB7A, both RAB4A and RAB5A had different behavior as the amount of RAB4A and RAB5A decreased, remained unchanged or increased in C13, A431 Pt and Hela Pt, respectively, compared to control cells (Figure S2). These results indicate that decreased expression of RAB7A and alterations of the late endocytic pathway are characteristics of chemoresistant cells and suggest a correlation between RAB7A function and chemotherapy response.

### 2.2. Modulation of RAB7A Abundance Influences the Chemoresistant Phenotype

**Figure 2:**
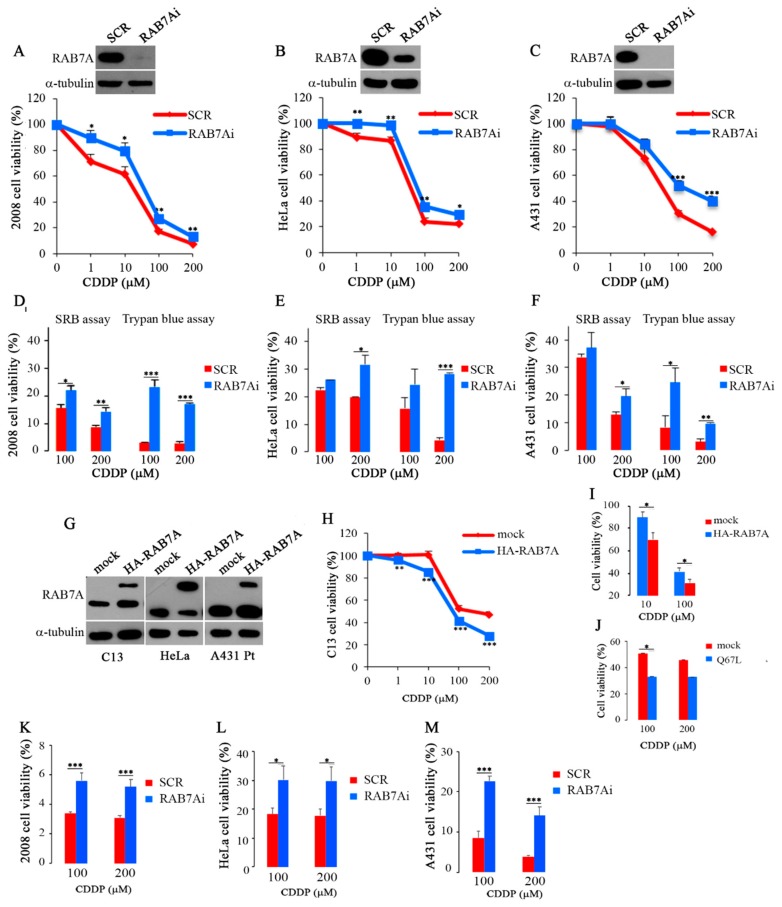
(**A**–**C**) MTT (3-(4,5-dimethylthiazol-2-yl)-2,5-diphenol tetrazolium bromide) and (**D**–**F**) Sulforhodamine B (SRB) and Trypan Blue (TB) assays were performed on control (SCR) and on RAB7Ai 2008, HeLa and A431 cells after treatment with cisplatin (CDDP) at the indicated concentrations. (**G**–**I**) C13 (mock) or C13 expressing HA-RAB7A cells, HeLa and A431 Pt control (mock) and expressing HA-RAB7A were either untreated or treated with CDDP at the indicated concentrations and cytotoxicity was analyzed with MTT assay. (**J**) C13 (mock) or expressing the HA-RAB7AQ67L mutant protein were either untreated or treated with CDDP for 24 h and subjected to MTT assay. (**K**–**M**) MTT assay was performed on control (SCR) and on RAB7Ai 2008, HeLa and A431 cells after treatment with CDDP for 48 h at the indicated concentrations. Each point represents mean ± SE of at least three independent experiments run in eight replicates. Statistical significance was measured by comparing values with values of control untreated cells. (* *p* ≤ 0.05, ** *p* ≤ 0.01 and *** *p* ≤ 0.001).

We then hypothesized that regulation of RAB7A abundance might have a role in the acquisition of the chemoresistant phenotype and, therefore, we wondered if chemoresistance could be actively induced by depleting RAB7A. 2008 cells were transfected with control RNA (SCR) or RAB7A-specific siRNA (RAB7Ai) and then treated with step-wise concentrations of CDDP or not treated for 24 h. Interestingly, we obtained a significant increase of resistance at all concentrations of CDDP in the RAB7A-depleted compared to control cells (Figure 2A). Similar results were obtained by silencing RAB7A in HeLa and A431 cells although to different extent (Figure 2B,C). These results were confirmed by Sulforhodamine B (SRB) and Trypan Blue assays (TB) (Figure 2D–F).

Then, we tested whether we could recover drug sensitivity in chemoresistant cells by increasing RAB7A expression. Wild-type HA-RAB7A was overexpressed in C13 cells (Figure 2G). CDDP treatment was performed 48 h after transfection and C13 cells transfected with empty vector (mock) were used as control. Interestingly, a change in the strong CDDP-resistant phenotype toward higher sensitivity to CDDP was observed with a significant decrease of the viability in C13 cells overexpressing HA-RAB7A versus control cells at all concentrations of the drug (Figure 2H).

Similar results were obtained after expression of HA-RAB7A in HeLa and A431 Pt cells (Figure 2G). Indeed, for instance, overexpression of RAB7A determined in treated (10 μM) HeLa cells a reduction of viability of about 30% while in mock HeLa cells the reduction of viability was about 10% (Figure 2I). Similarly, A431 Pt cells transfected with HA-RAB7A and treated with 100 μM CDDP showed a reduction of viability of about 70% while in mock transfected cells the treatment caused a reduction of viability of only about 60% (Figure 2I).

It is important to underline that differences in cisplatin sensitivity are not due to different proliferation rate between control cells and RAB7A manipulated cells. Indeed, first of all we have used as controls CDDP-treated RAB7A silenced and HA-RAB7A overexpressing cells. Also, we have already demonstrated that depletion of RAB7A does not influence cellular proliferation and viability in other cell lines [43]. Moreover, upon HA-RAB7A expression we observed no change in proliferation in A431Pt and HeLa cells but a significant increase of proliferation in C13 cells, further reinforcing our data on drug sensitization upon overexpression of RAB7A.

Half maximal effective concentration (EC50) values are used as a measure of drug’s potency. MTT (3-(4,5-dimethylthiazol-2-yl)-2,5-diphenol tetrazolium bromide) results obtained using C13, HeLa and A431 Pt cell lines overexpressing HA-RAB7A or mock and treated for 24 h with different concentrations of CCDP were used to measure EC50 values (Table 1). EC50 value of C13 expressing HA-RAB7 was about 67 μM while EC50 for mock C13 was 137 μM. Similarly, EC50 values of HeLa and A431 Pt expressing HA-RAB7A were 28 μM and 54 μM, respectively, while the values for mock HeLa and A431 Pt were 46 μM and 94 μM, respectively. EC50 values were calculated also for RAB7A-depleted cells. Significant differences between RAB7A-depleted cells and control cells were observed. Indeed, the EC50 value of 2008 RAB7A-depleted (RAB7Ai) cells was 88 μM, while EC50 for 2008 control cells was 47 μM. Similarly, EC50 values of RAB7A-depleted (RAB7Ai) HeLa and A431 cells were 78 μM and 100 μM, respectively, while the ones for control HeLa and A431 cells were 45 μM and 36 μM (Table 1).

Finally, in order to understand if sensitivity to CDDP was determined by RAB7A activity, we expressed in C13 cells the constitutively active RAB7AQ76L mutant. After treatment with CDDP, we observed that C13 cells became sensitive at both CDDP concentrations, suggesting that the active form of RAB7 is necessary (Figure 2J).

MTT assay was performed also on control (SCR) and on RAB7Ai 2008, HeLa and A431 cells after treatment with CDDP at 100 μM and 200 μM for 48 h and, consistently, we obtained a significant increase of resistance in RAB7A-depleted compared to control cells (Figure 2K–M).

Altogether, these data strongly indicate that modulation of RAB7A abundance influences chemoresistance as overexpression of RAB7A reduces CDDP-resistance while RAB7A silencing increases it.

### 2.3. RAB7A Depletion Determines CDDP Efflux in Extracellular Vesicles

**Figure 3:**
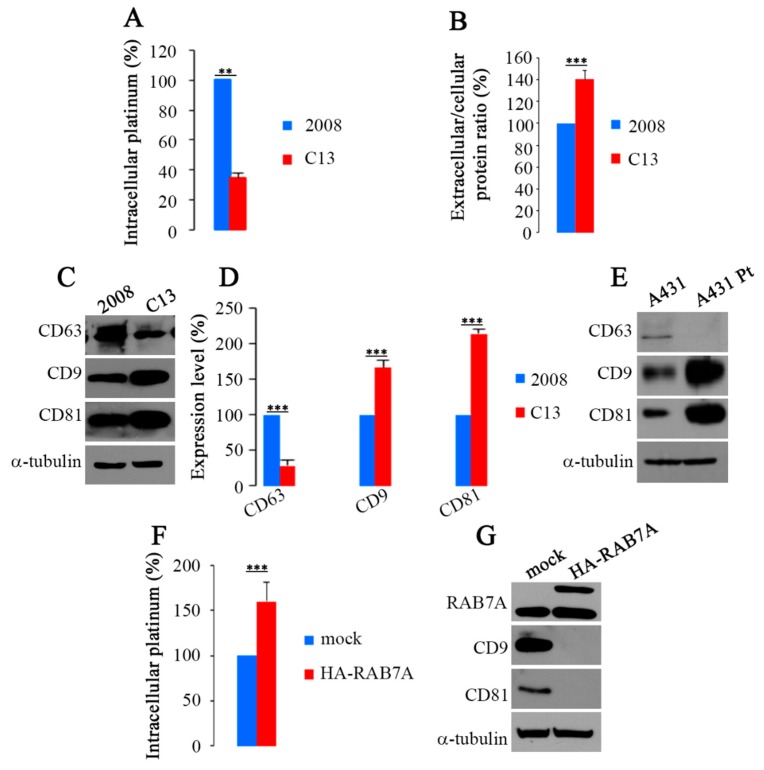
(**A**) Accumulation of platinum in 2008 and C13 cells was measured by Inductively Coupled Plasma-Atomic Emission Spectroscopy (ICP-AES) after treatment with 20 μM CDDP for 24 h. Obtained values were normalized on cell number. (**B**) Total amount of extracellular proteins released from 2008 and C13 cells normalized on total cell proteins. (**C,D**) Relative amounts of CD63, CD9 and CD81 were examined by WB and quantified. (**E**) Expression of CD63, CD9 and CD81 on extracellular vesicles (EVs) from A431 and A431 Pt cells. (**F**) Platinum accumulation in C13 (mock) or C13 expressing HA-RAB7A was measured by ICP-AES after 24 h of treatment with 20 μM CDDP. (**G**) Expression of CD9 and CD81 was evaluated on EVs from mock and HA-RAB7A transfected C13 cells. Data represent the mean ± SE. of at least three independent experiments. ** *p* ≤ 0.01 and *** *p* ≤ 0.001

In order to establish if chemoresistance is associated with decreased amount of CDDP in cells, we performed an ICP-AES analysis on cellular pellets after CDDP treatment (20 μM for 24 h). The results obtained confirmed that, as previously suggested [12], less CDDP is present inside C13 cells. Indeed, CDDP concentration in C13 cells was about 65% lower than in 2008 chemosensitive cells (Figure 3A).

Different kinds of EVs (Extracellular Vesicles) exist and they are released from many cell types, including cancer cells [44,45,46]. CDDP-resistant cells seem to export more CDDP compared to chemosensitive cells through exosomes, a type of EVs deriving from fusion MVBs (Multivesicular Bodies) with the plasma membrane, allowing to speculate that chemoresistance may be due to major exosomal export of CDDP [11,12]. Therefore, we isolated EVs from extracellular medium and analyzed the amount of extracellular proteins in 2008 and C13 cells. Interestingly, the amount of extracellular proteins was increased of about 40% in C13 versus 2008 cells (Figure 3B), suggesting an increased production of EVs. To establish which kind of EVs could be responsible for CDDP efflux we analyzed the EV extracts for the presence of CD63, CD9 and CD81 (Figure 3C). CD63, a tetraspanin protein, also known as LAMP3, is present on MVBs and on released exosomes but not in other EVs [44]. Analysis of C13 EVs revealed that CD63 expression was reduced of about 70% compared to 2008 control cells (Figure 3D). Instead, immunostaining of EV extracts with antibodies against CD9 and CD81, two proposed and validated general EV marker proteins [47], showed that C13 EVs displayed a strong increase of both CD9 and CD81 tetraspanins. Indeed, in EVs of C13 cells, the amount of CD9 and CD81 was increased of about 65% and 110%, respectively, compared to EVs of 2008 control cells (Figure 3D). Similarly, we observed reduced expression of CD63 and increased expression of both CD9 and CD81 tetraspanins in EVs of CDDP resistant A431 Pt cell line compared to A431 control cells (Figure 3E).

To check if RAB7A could be responsible for the differences observed between C13 and 2008 cells in terms of CDDP export, we overexpressed wild type-HA-RAB7A in C13 cells and monitored intracellular platinum. Interestingly, ICP-AES analysis revealed that expression of HA-RAB7A increased by about 60% the amount of intracellular platinum in these cells (Figure 3F).

Subsequently, to establish if modulation of RAB7A expression could affect EV production we analyzed the presence of the CD9 and CD81 markers in EVs in HA-RAB7A transfected C13 cells. Interestingly, abundance of CD9 and CD81 was strongly reduced upon RAB7A overexpression (Figure 3G). Altogether, these data suggest that chemoresistance is due to greater export of CDDP through EVs and that this mechanism is regulated by RAB7A.

### 2.4. Rab7A Expression and Response to Chemotherapy in Ovarian Cancer Patient Tissues

With the aim to investigate a possible correlation between RAB7A expression and chemotherapy response ex vivo, we have analyzed RAB7A expression in few tumor tissues of patients diagnosed with high grade serous ovarian cancer.

Epithelial ovarian cancer is one of the most lethal types of gynecological malignancies and the 5th leading cause of cancer-related mortality in females [48,49]. Despite enormous therapeutic improvements over the past years, the prognosis of these patients remains dismal and this is largely attributed to chemotherapeutic resistance following several cycles of treatment.

Tissues were obtained before and after chemotherapy treatment from patients subjected to neo-adjiuvant treatment. We hence performed a preliminar analysis of RAB7A protein levels on only 12 matched tumor tissues of patients for which both the naïve (pre-chemotherapy) and the post-chemotherapy residual tumor tissues were available within the Mitochondria in Progression of Endometrial and Ovarian cancers (MiPEO) study. Unfortunately, as it was extremely difficult to find and obtain samples from tissues of neo-adjuvant therapy-treated patients, we could analyze only a very low number of specimens considering also that this category of patients represents only 25–30% of high grade serous ovarian cancer cases [50]. Patient characteristics are shown in Table 2.

**Figure 4:**
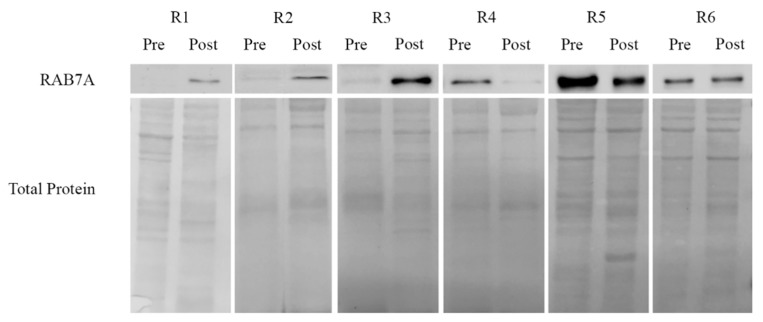
RAB7A expression was evaluated on protein extract from tissue tumor specimens of patients with ovarian cancer disease before (Pre) and after (Post) chemotherapeutic treatment. Levels of RAB7A protein was normalized on total Ponceau S protein staining.

Response to therapy was evaluated depending on different clinical and pathological parameters. Parameters as high number cycles of chemotherapy, not optimal debulked, low Surgical Complexity Score (SCS) and low Chemotherapy Response Score (CRS) were associated with a poor response to chemotherapy, therefore to chemoresistance. Based on the combination of these criteria, R1, R2 and R3 were considered to be poor responders. R6 was evaluated as partial responder (6 cycles of chemotherapy administered, complete cytoreduction after debulking surgery, high complexity of the surgical approach, low histological CRS); R4 and R5 were considered as complete responders (complete cytoreduction after debulking surgery, low number of cycles administered, low SCS and intermediate CRS score). As shown in Figure 4, we observed reduced expression of RAB7A in pre-chemo cancer samples from R1, R2 and R3 compared to matched post-chemo tissue, while increased RAB7A levels were displayed by patients with complete response to therapy, such as R4 and R5. No variations were observed in R6, who only showed a partial response to drugs.

With the limitation of the extremely low number of samples, these data suggest that RAB7A expression in pre-chemotherapy cancer tissues may predict response, although studies are warranted on a larger cohort of patients in order to draw definitive conclusions.

## 3. Discussion

In this paper, we showed that downregulation of RAB7A, determining alterations in the late endocytic pathway and lysosomal defects with dysfunctional CDDP sequestration, influences chemoresistance, through the increase of EV-mediated CDDP efflux. Chemoresistance is a cogent issue in clinical oncology representing the major obstacle for successful therapy in many types of treated cancers. Various mechanisms determine innate or acquired chemoresistance in cancer cells and, among these, reduced cellular uptake, ATP-driven drug efflux from cells, quantitative and qualitative alterations in target proteins, drug compartmentalization, drug metabolism as well as apoptosis evasion [54]. Recently, defects in lysosomal function have been implicated in drug resistance. In fact, it was demonstrated that lysosomes are able to sequestrate multiple hydrophobic weak-base chemotherapeutic agents through passive ion trapping-based transport, reducing accessibility of these drugs to their targets’ sites [55]. Moreover, lysosomal trapping may determine T-cell Transcription Factor EB (TFEB)-mediated lysosomal biogenesis resulting in an enlarged lysosomal compartment, capable of enhanced drug sequestration [56]. In addition to passive drug sequestration of hydrophobic weak base chemotherapeutics, other mechanisms of lysosome-mediated drug resistance have also been reported; these include active lysosomal drug sequestration mediated by ATP-driven transporters from the ABC superfamily, and a role for lysosomal copper transporters in cancer resistance to platinum-based chemotherapeutics [57,58]. Thus, lysosomes play an important role in detoxification of heavy metals, including platinum, but it was also hypothesized that sequestration of CDDP into lysosomes, rather than serving as detoxification pathway, may also induce lysosomal damage and apoptosis [57,58]. In fact, lysosome-dependent cell death represents a form of regulated cell death initiated primarily by lysosomal membrane permeabilization (LMP) [59]. LMP involves relocation of lysosomal constituents into the cytosol, which, in turn, triggers a whole cascade of events leading to cell death [59]. Therefore, it has been speculated that impairment of the lysosomal compartment, determining reduced CDDP sequestration, protects cells from LMP and induces the chemoresistance phenotype [12].

It has been demonstrated that CDDP accumulates in lysosomes that are reduced in size and number in chemoresistant cells suggesting that transporter expression and subcellular localization, rather than lysosome number, is the limiting factor in lysosome-mediated CDDP resistance [11,12,14,60]. In addition, it was reported that CDDP resistant cells were characterized by incomplete degradation of internalized Epidermal Growth Factor (EGF) and slow processing of lysosomal cysteine protease cathepsin L [14]. Furthermore, treatment with vacuolar proton pump inhibitors, such as Bafilomycin A1, mimics carboplatin resistant phenotype in sensitive cells, again suggesting that reduced endocytosis and lysosomal defects of chemoresistant cells could contribute to drug resistance [14].

Here, analyzing the lysosomal compartment, we confirmed and extended previous data, observing a strong reduction in the number, size and acidification of lysosomes in chemoresistant cells. Considering the pivotal role of RAB7A in the late endocytic pathway and, in particular, in lysosomal biogenesis and function [18,19], we investigated whether the alterations described as characteristic of chemoresistant cells could be due to altered RAB7A function. In agreement with our hypothesis, we have found reduced RAB7A expression in CDDP resistant cells while no common trend of expression was revealed for RAB4A and RAB5A and, consistently, it was previously demonstrated that CDDP is not or is barely detectable in RAB4A- and RAB5A-positive early endosomes [11,12]. The mechanism for RAB7A reduction does not seem to involve transcriptional regulation, as RT-PCR revealed no changes in the amount of mRNA in chemoresistant and chemosensitive cells. Thus, one possibility is that, in chemoresistant cells, RAB7A is subjected to proteasomal degradation. However, it has been described that decrease in RAB7A ubiquitination causes a reduction of RAB7 amounts due to protein stability loss and RAB7A stability is normally guaranteed by interaction with the RAB7 effector RILP [61]. Thus, it is also possible that reduction in RAB7A protein amounts is caused by decreased ubiquitination.

Downregulation of RAB7A in CDDP-resistant cells was accompanied by decreased expression of LAMP1 and of ATP6V1G1, a subunit of the vacuolar ATPase, indicating alterations at the level of late endosomes and/or lysosomes that could be due to downregulation of RAB7A. Indeed, RAB7A is important for transport to lysosomes but also for lysosomal acidification through the ATP6V1G1 subunit of V-ATPase pump that is regulated by the RAB7A effector RILP [29].

Dysfunction of lysosomal acidifications was previously correlated with anti-cancer drug lysosomal accumulation and enhanced exocytosis [62]. Indeed, lysosomal exocytosis was shown to be induced by disruption of lysosomal acidity, achieved either by treatment with the vacuolar proton pump inhibitor Bafilomycin A1, or with lysosome-accumulating weak amines [63]. Thus, the lower number of lysosomes in CDDP resistant cells might be due to enhanced lysosomal exocytosis.

Importantly, we were able to induce chemoresistance in chemosensitive cells by silencing RAB7A while reduction of chemoresistance was observed upon RAB7A overexpression thus indicating that the expression levels of RAB7A are directly correlated to chemoresistance.

What is the mechanism by which RAB7A modulates chemoresistance? We have analyzed accumulation of CDDP in chemoresistant versus chemosensitive cells and found that, as expected, the amount of CDDP was lower in chemoresistant cells and thus we hypothesized increased efflux through EVs. Among EVs, ectosomes derive from shedding of vesicles from the plasma membrane, while exosomes are formed by intraluminal vesicles that are released upon exocytosis of MVBs [44]. RAB7A is present on MVBs together with CD63, a specific marker of exosomes, while CD9 and CD81 are general markers of EVs [44]. In particular, because of inconsistence of results obtained with other markers, CD9 and C81 were defined as the best markers for assessing abundance of EVs [47]. The role of RAB7A in exosome release is still not clear [64]. In fact, high expression of RAB7A has been correlated with high production of exosomes in melanoma cell lines [65] and a RAB7A-dependent exosomal pathway has been demonstrated for miR-143 export [66]. However, RAB7A silencing decreased exosomal release in MCF7 cells [67] but did not have any effect on exosomal secretion in HeLa B6H4 tumor cells [68]. Recently, it has also been demonstrated that ovarian cancer cells in hypoxic conditions increased their exosome release by upregulating RAB27A and downregulating several protein among which RAB7A, and also by promoting a more secretory lysosomal phenotype with increased CDDP efflux [69].

According to the data indicating that RAB7A silencing decreases exosomal secretion [67], we observed a strong decrease in CD63 staining of EVs purified from chemoresistant cells that display low levels of RAB7A expression, although the total amount of extracellular extracts (and therefore of EVs) but also the abundance of CD9 and CD81 was increased. Altogether, these data indicate that lower RAB7A expression increases the total amount of EVs while exosomes are reduced. Consistently, our data indicate that modulation of RAB7A expression affects abundance of exosomes and EVs, possibly influencing their formation and/or release.

Importantly, RAB7A seems to be involved also in the delivery of CD9-positive EV-associated material, once captured by the targeted cells, to the nucleus, as subdomains of RAB7A-positive late endosomes and subdomains of the nuclear envelope create a new compartment with this function [70]. Therefore, it is also possible to envisage that decrease of RAB7A expression inhibits this delivery possibly blocking upstream uptake of EVs by cells and thus leading to accumulation of EVs in the extracellular medium.

**Figure 5:**
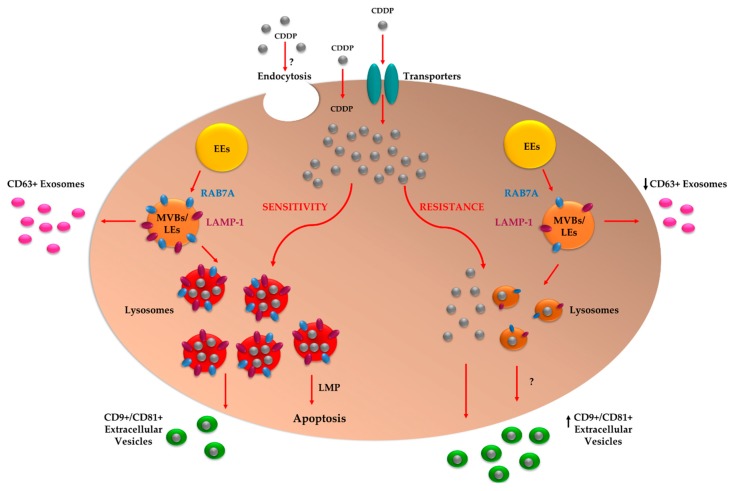
Proposed chemoresistance mechanism mediated by RAB7A. CDDP enters the cell through specific transporters, by passive diffusion or by endocytosis. The uptake of CDDP in cells with functional endocytic trafficking leads to activation of apoptosis following sequestration of drug in lysosomes and consequent Lysosomal Membrane Permeabilization (LMP) induction. Uptake of CDDP into cells in which RAB7A is downregulated with a reduction in the size, number and acidification of lysosomes, leads to CDDP efflux through the EVs and then to a resistant phenotype. EE: Early Endosome; LE: Late Endosome; MVB: Multivesicular Body.

In conclusion, defects in lysosomes resulting from RAB7A downregulation in chemoresistant cells could determine alterations in CDDP sequestration resulting in increased CDDP efflux, that could be mediated by EVs, causing the chemoresistant phenotype (Figure 5). However, exactly how RAB7A influences the formation of EVs remains to be established.

With the attempt to translate our molecular findings into clinics, we exploited a very low number of cases of high grade serous ovarian carcinomas, a type of gynecological cancer in which the issue of chemoresistance is a cogent one, as a relatively high number of patients do not respond to the standard lines of chemotherapy, namely a combination of platinum derivatives and taxanes [71,72]. We selected our cases on the basis of the availability of cancer tissues before and after the treatment, and of the completeness of the corresponding clinical data.

Despite the extremely low number of cases here preliminarily analyzed, the correlation between RAB7A protein levels and the status of responders versus non-responders (or partial responders) was coherent with the results obtained in cell lines. Indeed, in responders the decrease in RAB7A may indicate an ongoing selection of the clones that have a lower protein level, as they would be fitter to resist to chemotherapy. In this frame, it will be interesting to observe whether any potential relapse may occur in these patients starting from low-RAB7A cells within the post-chemotherapy mass. In non-responders, we initially observe nearly undetectable levels of RAB7A. Surprisingly, a high number of chemotherapy cycles (which is one of the marker to define a patient as poor responder) resulted into an increase of RAB7A in post-chemo cancers. Although this result may appear to be in disagreement with our data, interestingly two out of three patients showed an uncommon longer OS (Table 2) compared with the median OS reported in the literature for patients with these features (25 months [73]). Whether such more favorable chemotherapeutical outcome depends on RAB7A increase warrants investigation, and the mechanism of selection of clones with higher RAB7A levels after chemotherapy in poor responders patients may suggest oncojanus function for this GTPase [74,75]. In fact, it is important to refer that the role of Rab7 in cancerous events is still not completely clear as both pro-tumorigenic and oncosuppressor functions of Rab7 have been described [18]. In light of this, Rab7 could have dose- and tumor-type-dependent roles in cancer cell proliferation and invasion, defining its oncojanus function [74]. Accordingly, low levels of RAB7 were described in benign nevi of melanoma, then increased expression was observed during cancer development while subsequent downregulation was associated to cancer progression in order to favor the invasive phenotype and to switch to metastatic stages [76]. Clearly, however, clinical studies involving a higher number of tumor specimens are required to draw definitive conclusion and to investigate this issue in more details.

## 4. Materials and Methods

### 4.1. Cells Lines

HeLa cells (human cervix carcinoma) and their CDDP-resistant counterpart (HeLa Pt) were grown in DMEM (Sigma-Aldrich, St. Louis, MO, USA) while 2008 (human cervical cancer cells), A431(human cervix squamous carcinoma) and their CDDP-resistant sublines C13 and A431 Pt were maintained in RPMI-1640 medium (Sigma-Aldrich). Media were supplemented with 10% FBS, 2 mM glutamine, 100 U/mL penicillin and 10 mg/mL streptomycin (Sigma-Aldrich). Cells were grown in a 5% CO_2_ incubator at 37 °C. HeLa cells were purchased from the ATCC (Manassas, VA, USA); 2008 and C13 cells were a kind gift from G. Marverti (University of Modena and Reggio Emilia, Modena, Italy) [77]; A431 and A431 Pt cells were a kind gift from M. Montopoli (University of Padua, Padua, Italy) [78].

### 4.2. Case Series

The study was performed on 12 cases of patients diagnosed at the S. Orsola-Malpighi Hospital, (Bologna, Italy) with high grade serous ovarian cancer, FIGO stage IIIC-IVA, not cytoreducible in up front at primary surgery. All subjects gave their informed consent for inclusion before they participated in the study. The study was conducted in accordance with the Declaration of Helsinki and the protocol was approved by the Independent Ethics Committee of S. Orsola-Malpighi Hospital (107/2011/U/Tess of MiPEO studies). An alpha-numeric code (from R1 to R6) was assigned to the cases. Tumor stage was determined according to the International Federation of Gynecology and Obstetrics guidelines (FIGO). All patients underwent debulking surgery after three or more cycles of chemotherapy depending on the response to therapy.

### 4.3. Tumors Specimens

All samples were fresh frozen. Haematoxylin and eosin sections were done to identify tumor areas. Pre-chemo specimens were obtained with laparoscopic biopsy at the moment of diagnosis, post-chemo specimens were obtained after chemotherapy with carboplatin and paclitaxel, during the debulking surgery.

### 4.4. Induction of Chemoresistance in HeLa Cells

HeLa cells were treated with low concentration of CDDP (1–5 µM) for 6 months to obtain their resistant counterpart HeLa Pt. This treatment was performed alternating weekly CDDP exposure and recovery with drug-free medium. Acquisition of chemoresistance was tested through MTT assay after step-wise treatment with CDDP for various time points (Figure S3).

### 4.5. Transfection and RNA Interference

Transfection and silencing of HeLa cells was performed as described [29]. The siRNA used are indicated in Table S1. Transfection of C13 and A431 Pt cells was performed using Amaxa Cell Line Nucleofector Kit V (Lonza, Basel, Switzerland) following manufacturer’s instructions.

### 4.6. Western Blotting

Cells were lysed in Laemmli Buffer (100 mM Tris–HCl, pH 6.8, containing 4% SDS, 20% glycerol and 0.2% blue bromophenol). Tissue (~200 mg) were homogenized in RIPA Buffer (Tris-HCl pH 8 50 mM, NaCl 150 mM, NP40 1%, sodium deoxycholate 0.5%, SDS 0.1%), with freshly added protease inhibitors, using a homogenizer (Qiagen Tissue Ruptor, Hilden, Germany). Western blotting (WB) was performed as described [29]. Antibodies against RAB7A, RAB5A, RAB4A, ATP6V1G1, CD81 were from Santa Cruz Biotechnology (Dallas, TX, USA), antibody against LAMP1 (H4A3) was deposited to the Developmental Studies Hybridoma Bank (University of Iowa, Iowa City, IA, USA) by J.T. August and J.E.K. Hildreth while anti-α-tubulin was from Sigma-Aldrich. Secondary anti-mouse and anti-rabbit antibodies, conjugated with HRP, were from Invitrogen (Carlsbad, CA, USA) and Santa Cruz Biotechnology (Dallas, TX, USA), respectively. Protein levels by WB were quantified by densitometry using ImageJ software (Version 1.5Oi, Bethesda, MD, USA) normalizing against α-tubulin. For tissue extracts, the staining of nitrocellulose membrane with Ponceau S (Sigma-Aldrich, Saint Louis, MO, USA) was used as loading control.

### 4.7. Quantitative Real Time PCR

RNA extraction and quantitative Real Time PCR (qRT-PCR) were performed as described previously [29]. The primers used are reported in Table S1.

### 4.8. Immunofluorescence and Live Microscopy

Cells grown on 11 mm round glass coverslips were permeabilized, fixed and incubated with antibodies as described previously [29]. Subsequently, cells were imaged with an LSM 700 confocal microscope (Zeiss, Oberkochen, Germany). For live microscopy cells were seeded into microscopy chambers (8 well μ-slide, Ibidi GmBh, Martinsried, Germany) and incubated with LysoTracker Red DND-99, LysoSensor Blue DND-167, LysoSensor Yellow/Blue DND 160 and CellMask Green (ThermoFisher Scientific, Carlsbad, CA, USA) as described previously [79]. Intensity of fluorescence and size of lysosomes were determined by ImageJ software (Version 1.5Oi, Bethesda, MD, USA).

### 4.9. Cell viability Measurements

Cell viability was assayed by MTT (3-(4,5-dimethylthiazol-2-yl)-2,5-diphenol tetrazolium bromide), Sulforhodamine B (SRB, Sigma-Aldrich, Saint Louis, MO, USA) or Trypan Blue (TB, Sigma-Aldrich, Saint Louis, MO, USA). Conversion of MTT by mitochondrial succinate dehydrogenase was used as an indicator of cell viability as described [80]. The SRB assay, based on cellular protein content, was performed as described [81]. For TB assay, live and dead cells were counted with an optical microscope after staining. For MTT and SRB assay, plates reading was performed on a Multilabel Plate Reader (Victor X5, PerkinElmer, Waltham, MA, USA) at 570 nm. EC50 was calculated after 24 h as the concentration giving a 50% decrease in the cell number compared to untreated cells using the GraphPad Prism software (Version 6.0, GraphPad, San Diego, CA, USA).

### 4.10. Inductively Coupled Plasma-Atomic Emission Spectroscopy (ICP-AES) Analysis

For the determination of platinum concentration, samples were previously heat treated with 0.5 mL of 67% ultrapure nitric acid and 0.2 mL of hydrogen peroxide to obtain a clear solution. Samples were then diluted to a final volume of 5 mL, to obtain a suitable concentration for the acid used in the mineralization process and avoid damage to the analytic system. Samples were filtered before analysis through syringe filters (0.45 μm) to prevent entry of any remaining suspension in the measuring instrument.

### 4.11. Extracellular Vesicles (EVs) Purification

Cells were washed with PBS and incubated with medium containing 10% exosome-free FBS for 24 h. Conditioned medium was collected and centrifuged at 300 g for 10 min at 4 °C to pellet cells. Supernatant was collected and centrifuged at 16,500 g for 20 m in at 4 °C to pellet apoptotic bodies and cell debris. Supernatant was then filtered through 0.22 μm filters and ultracentrifuged at 110,000 g for 70 min at 4 °C. Pellets were resuspended in PBS, ultracentrifuged at 110,000 g for 70 m in at 4 °C and resuspended in RIPA lysis buffer centrifuged for 10 min at 13,400 g and supernatant was collected. In parallel, lysates from cell monolayers were prepared using RIPA buffer. Protein quantification was performed using the Bradford assay.

### 4.12. Statistical Analysis

All experiments were repeated at least three times and represented as mean ± standard error (SE). Statistical significance was determined for all experiment through Student’s *t* test for unpaired data (* *p* ≤ 0.05, ** *p* ≤ 0.01 and *** *p* ≤ 0.001).

## 5. Conclusions

In summary, we have elucidated a new RAB7A-dependent resistance mechanism to CDDP and demonstrated that it is possible to alter drug response by modulating RAB7A expression. RAB7A overexpression might indeed restore normal CDDP trafficking inducing chemosensitivity, which may have extensive implications for future identification of drugs to upregulate of RAB7A in order to improve therapeutic response to platinum compounds.

## Figures and Tables

**Table 1:** Cisplatin (CDDP) EC_50_ values in RAB7A overexpressing and silenced cell lines.

Cell Lines	EC50 (µM)
C13 mock C13 HA-RAB7A	137 μM ± 0.064 (***) 67 μM ± 0.057
2008 SCR 2008 RAB7Ai	47 μM ± 0.046 (*) 88 μM ± 0.063
A431 Pt mock A431 Pt HA-RAB7A	94 μM ± 0.06 (**) 54 μM ± 0.056
A431 SCR A431 RAB7Ai	36μM ± 0.138 (*) 100 μM ± 0.118
HeLa mock HeLa HA-RAB7A	46 μM ± 0.049 (*) 28 μM ± 0.072
HeLa SCR HeLa RAB7Ai	45 μM ± 0.046 (***) 78 μM ± 0.039

EC50: half maximal effective concentration; * *p* ≤ 0.05, ** *p* ≤ 0.01 and *** *p* ≤ 0.001.

**Table 2:** Cases and clinical features.

Patients	Age at Diagnosis	Stage (FIGO)	Chemotherapy (*No.* of Cycles)	SCS [51]	CC [52]	CRS [53]	PFS	OS	Follow-up Status
R1	32	IIIC	6	10	2	NA	14	32	DOD
R2	77	IIIC	6	2	1	NA	45	69	DOD
R3	75	IIC	7	2	2	NA	15	32	DOD
R4	61	IVA	6	3	0	NA	71	71	AWOD
R5	68	IVA	3	10	0	2	10	20	DOD
R6	44	IVA	6	10	0	1	25	25	AWOD

SCS: Surgical complexity score, SCS groups: low = 3 or fewer, intermediate = 4–7, high = 8 or more; CC: Completeness of cytoreduction, CC groups: no disease = 0, disease present up to 0.25 cm = 1, disease present 0.25–2.5 cm = 2, disease greater than 2.5 cm = 3; CRS: Chemotherapy response score, CRS groups: no or minimal tumor response = 1, appreciable tumor response = 2, Complete or near-complete response = 3; NA:. Not available; PFS: Progression Free Survival; OS: Overall Survival; DOD: Dead Of Disease; AWOD: Alive Without Disease.

## Supplementary Materials

The following are available online at http://www.mdpi.com/2072-6694/11/1/52/s1, Figure S1: Late endocytic pathway in A431/A431 Pt and HeLa/HeLa Pt cell line, Figure S2: Expression of endocytic Rabs in chemosensitive and chemoresistant cell lines, Figure S3: Test of acquired chemoresistance of HeLa Pt by MTT assay; **Table S1**: Sequences of primers and siRNA used in this study.
