# Supplementary material for: Modulation of RAB7A Protein Expression Determines Resistance to Cisplatin through Late Endocytic Pathway Impairment and Extracellular Vesicular Secretion
Source: Cancers (Basel). 2019 Jan 8;11(1):52. doi: 10.3390/cancers11010052 (PMC6357196; doi:10.3390/cancers11010052)
Supplement: Supplementary file 1 [file cancers-11-00052-s001.pdf]

Supplementary Materials: Modulation of RAB7A protein expression determines resistance to cisplatin through late endocytic pathway impairment and extracellular vesicular secretion

Flora Guerra, Aurora Paiano, Danilo Migoni, Giulia Girolimetti, Anna Myriam Perrone, Pierandrea De Iaco, Francesco Paolo Fanizzi, Giuseppe Gasparre and Cecilia Bucci

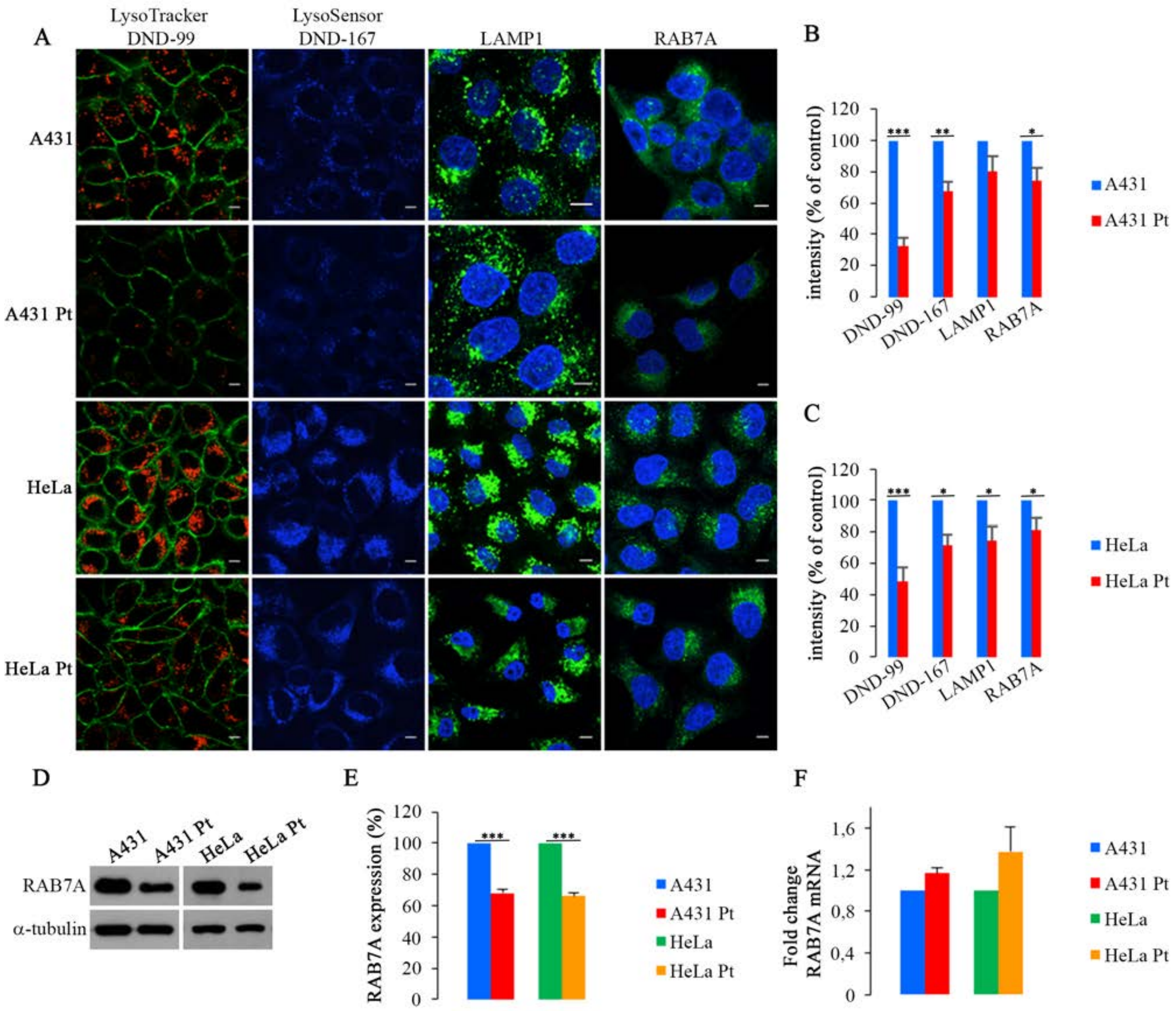

Figure S1. Late endocytic pathway in A431/A431 Pt and HeLa/HeLa Pt cell lines. (**A**) A431, A431 Pt, HeLa and HeLa Pt cells were labeled live with Lysotracker Red DND-99 and Cell Mask (green) and with LysoSensor DND-167 (blue) or fixed and immunostained with anti-LAMP1 and anti-RAB7A antibodies. Nuclei were stained with DAPI (blue). Scale bar: 10 µm. (**B,C**) IF intensity quantification in A431/A431Pt andHeLa/HeLa Pt cells. (**D,E**) Relative amounts of RAB7A were detected in A431/A431 Pt and HeLa/HeLa Pt cells by WB and quantified. (**F**) qRT-PCR to quantify RAB7A mRNA. Data represent the mean ± SEM of at least three independent experiments. * $p \leq 0.05$, ** $p \leq 0.01$ and *** $p \leq 0.001$

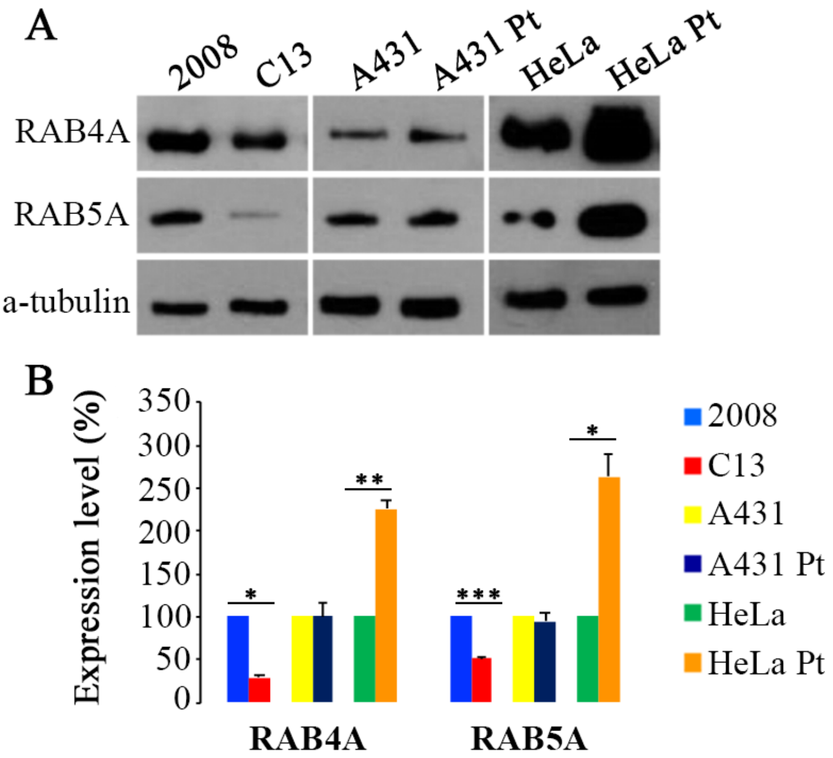

Figure S2. Expression of endocytic Rabs in chemosensitive and chemoresistant cell lines. (**A**) Relative amounts of RAB4 and RAB5 were detected in 2008/C13, A431/A431 Pt and HeLa/HeLa Pt cells by WB and quantified though densitometric analysis with ImageJ software (**B**). Values are the mean ± SEM of at least three experiments and are expressed as percentage of control. * $p \leq 0.05$, ** $p \leq 0.01$ and *** $p \leq 0.001$.

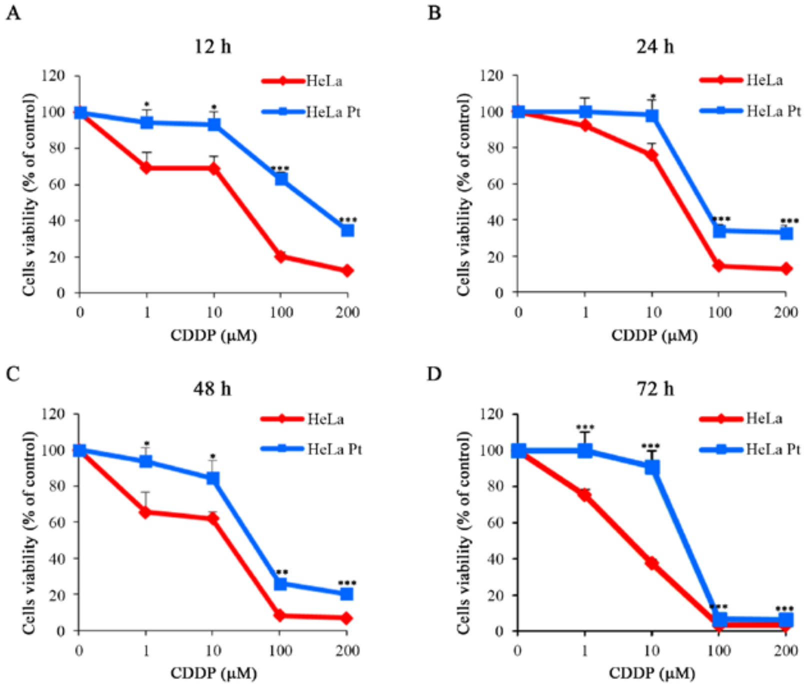

Figure S3. Test of acquired chemoresistance of HeLa Pt by MTT assay after treatment with 0, 1, 10, 100 and 200 µM of CDDP for 12 h (**A**), 24 h (**B**), 48 h (**C**) and 72 h (**D**). Each point represents mean ± SEM of at least three independent experiments run in eight replicates. Statistical significance was measured by comparing values with values of control untreated cells (* $p \leq 0.05$, ** $p \leq 0.01$ and *** $p \leq 0.001$). Percentage of viability after treatment resulted significantly higher at all concentrations and all times compared to HeLa cells, used as control.

Table S1. Sequences of primers and siRNA used in this study.

Name		Sequence
**RAB7A**	Forward	5′-CACAATAGGAGCTGACTTTCTGACC-3′
	Reverse	5′-GTTCCTGTCCTGCTGTGTCCCATATC-3′
**GAPDH**	Forward	5′-GGTGGTCTCCTCTGACTTCAACA-3′
	Reverse	5′-GTTGCTGTAGCCAAATTCGTTGT-3′
**RAB7A siRNA**	Sense	5′-GGAUGACCUCUAGGAAGAATT-3′
	Antisense	5′-UUCUUCCUAGAGGUCAUCCTT-3′
**Control RNA**	Sense	5′-ACUUCGAGCGUGCAUGGCUTT-3′
	Antisense	5′-AGCCAUGCACGCUCGAAGUTT-3′

© 2019 by the authors. Submitted for possible open access publication under the terms and conditions of the Creative Commons Attribution (CC BY) license (http://creativecommons.org/licenses/by/4.0/).
